# Eosinophils from Physiology to Disease: A Comprehensive Review

**DOI:** 10.1155/2018/9095275

**Published:** 2018-01-28

**Authors:** Giuseppe A. Ramirez, Mona-Rita Yacoub, Marco Ripa, Daniele Mannina, Adriana Cariddi, Nicoletta Saporiti, Fabio Ciceri, Antonella Castagna, Giselda Colombo, Lorenzo Dagna

**Affiliations:** ^1^Università Vita-Salute San Raffaele, Milan, Italy; ^2^Unit of Immunology, Rheumatology Allergy and Rare Diseases, IRCCS Ospedale San Raffaele, Milan, Italy; ^3^Unit of Infectious Diseases, IRCCS Ospedale San Raffaele, Milan, Italy; ^4^Unit of Haematology, IRCCS Ospedale San Raffaele, Milan, Italy

## Abstract

Despite being the second least represented granulocyte subpopulation in the circulating blood, eosinophils are receiving a growing interest from the scientific community, due to their complex pathophysiological role in a broad range of local and systemic inflammatory diseases as well as in cancer and thrombosis. Eosinophils are crucial for the control of parasitic infections, but increasing evidence suggests that they are also involved in vital defensive tasks against bacterial and viral pathogens including HIV. On the other side of the coin, eosinophil potential to provide a strong defensive response against invading microbes through the release of a large array of compounds can prove toxic to the host tissues and dysregulate haemostasis. Increasing knowledge of eosinophil biological behaviour is leading to major changes in established paradigms for the classification and diagnosis of several allergic and autoimmune diseases and has paved the way to a “golden age” of eosinophil-targeted agents. In this review, we provide a comprehensive update on the pathophysiological role of eosinophils in host defence, inflammation, and cancer and discuss potential clinical implications in light of recent therapeutic advances.

## 1. Introduction

### 1.1. Definitions

Eosinophils represent up to 6% of the bone marrow resident nucleated cells and are routinely measured as part of the full blood cell count. When eosinophil absolute count exceeds 450–500 cells/*μ*l the term eosinophilia applies. A threshold of 1500 cells/*μ*l is usually employed to define blood hypereosinophilia. The association of blood hypereosinophilia with established eosinophil-related organ damage, in the absence of other potential confounders, defines a hypereosinophilic syndrome (HES), whereas clinically silent cases are usually termed hypereosinophiliae of undetermined significance (HEUS). The term primary (or intrinsic) hypereosinophilia refers to the presence of an overt haematological malignancy or proliferative disorder characterised by neoplastic eosinophils as the cause of the disease. Secondary (or extrinsic) hypereosinophiliae comprise all cases in which eosinophil proliferation is stimulated by other (at least in part) known causes such as lymphoid malignancies, parasitic or inflammatory disorders. Idiopathic hypereosinophilia possibly constitutes a provisional category that includes all cases in which a clear underlying aetiology cannot be identified [1].

### 1.2. Eosinophil Dynamics across the Human Body

**Figure 1:**
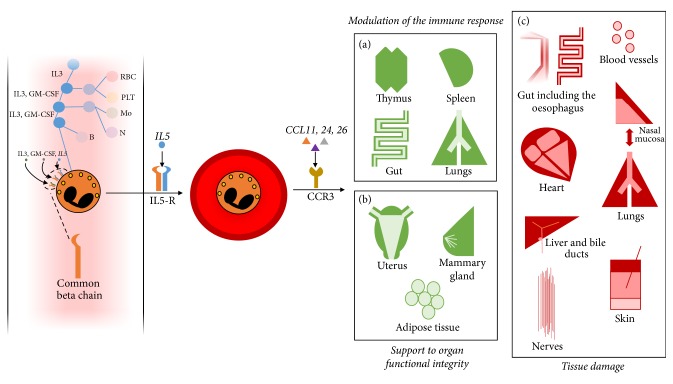
*Eosinophil dynamics. *Eosinophil differentiate in the bone marrow under stimulation by IL3, GM-CSF, and IL5, which bind to receptors sharing a common beta chain. Either the beta chain or the specific partner chains of these receptors constitute potential targets for pharmacological modulation. IL5 is crucial for the last stage of eosinophil maturation in the bone marrow as well as for eosinophil release in the circulating blood and subsequent survival. An array of chemokines targeting the chemokine receptor CCR3 promote eosinophil recruitment into organs and tissues. A first set of target tissues hosts a population of regulatory eosinophils involved in the maintenance of the immune homeostasis (a) or of organ functional integrity (b). Other tissues (such as the heart, the gut including the oesophagus, the respiratory tract, the skin, the liver, and bile ducts as well as central or peripheral nerves) are instead targets for eosinophil infiltration during inflammation (c). Eosinophils also promote intravascular inflammation and are able to trigger the coagulation cascade.

Eosinophil development and maturation occur in the bone marrow over approximately a week under exposure of myeloid precursors to IL3, GM-CSF, and IL5. The latter is of particular relevance for the final stage of eosinophil differentiation and as a trigger to eosinophil migration into the circulating blood (Figure 1). Furthermore, IL-5 is a key cytokine in the survival and persistence of circulating and tissue eosinophils, preventing apoptosis and promoting cell activation. CD34+ progenitor cells, group 2 innate lymphoid cells (ILC-2), Th2 lymphocytes, invariant natural killer T cells, and mast cells are major sources of IL5 [2, 3]. In addition, IL5 can be released by eosinophils in an auto/paracrine manner [4–7]. Chemokines such as CCL11, CCL24, and CCL26 (also known as eotaxin 1, 2, and 3, resp.) eventually promote eosinophils recruitment into tissues within 8–12 hours since their release from the bone marrow [8]. The chemokine receptor CCR3 plays a crucial role to this purpose, since it binds to all three eotaxins as well as to other inflammatory stimuli such as CCL5, CCL7, and CCL13.

Under physiological conditions, eosinophils are detectable in several organs, where they exert a wide range of homeostatic tasks. Basal levels of eosinophils are regulated by ILC-2 activity, which in turn responds to variations in energy intake and to circadian rhythms [2]. Eosinophils infiltrate primary and secondary lymphoid organs such as the thymus, the lymph nodes, and the spleen as well as Peyer's patches within the gut, possibly assisting other immune cells in their maturation and homing [9] (Figure 1). Eosinophils promote plasma cell survival within the bone marrow and the gut [9–11] and ensure a physiological balance between T-helper and T-regulatory responses in the gut and in the lungs [12, 13]. Moreover, they are able to shape the characteristics of the immune response by performing antigen presentation [5, 14, 15]. Besides immunomodulatory functions, eosinophils also support the functional integrity of nonlymphoid organs such as the adipose tissue (where they control glucose tolerance, preventing obesity) and are required for the optimal development of the mammary gland. Eosinophils are also detectable in the normal uterus, although their putative homeostatic role in that setting is less clear [15] (Figure 1). Finally, eosinophils can produce several growth factors, thus potentially contributing to tissue repair [5, 16].

**Figure 2:**
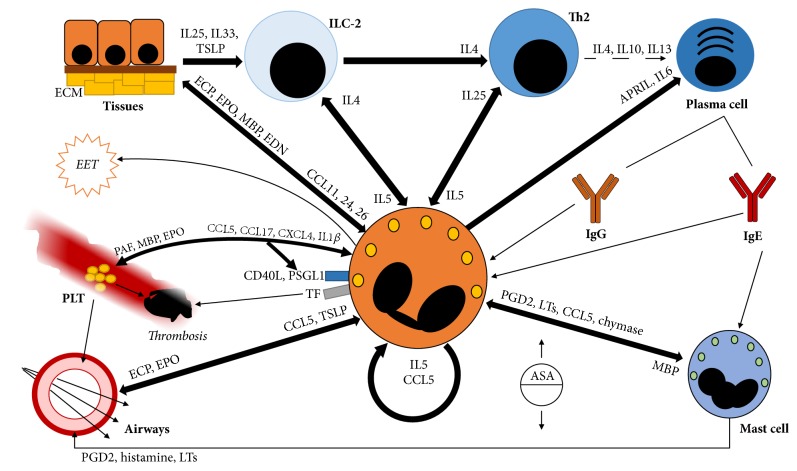
*Eosinophil interactions with cells and tissues.* Eosinophils are part of a complex network of signalling molecules and exert a wide range of behaviours towards interacting cells and tissues. Bidirectional cytokine signalling favours the reciprocal activation of group 2 innate lymphoid cells (ILC-2) and eosinophils, Th2 cells, and eosinophils as well as mast cells and eosinophils. ILC-2 are a major source of IL5 for eosinophils, which in turn can maintain ILC-2 activation through the release of IL4. ILC-2 play also a pivotal role in the cross-talk between tissues and inflammatory cells, as they respond rapidly to tissue-derived IL25, IL33, and thymic stromal lymphopoietin (TSLP) and promote Th2-responses by secreting IL4. Th2 cells favour eosinophil activation and survival by releasing an array of moieties, primarily IL5. Eosinophils in turn are able to sustain Th2 responses through the production of IL25. Downstream Th2 cells, eosinophils contribute to the humoral adaptive response by releasing plasma cell survival factors such as IL6 or A proliferation inducing ligand (APRIL) and by recognising class G and class E immunoglobulin through their surface receptors. Mast cells respond to the release of eosinophil-derived MBP and are major triggers of acute inflammation under several inflammatory conditions. In addition, they promote eosinophil activation by releasing prostaglandins such as prostaglandin D2 (PGD2), chemokines such as CCL5, and leukotrienes. Leukotrienes are well-known mediators of acute and chronic airways inflammation. Thus, not surprisingly, aspirin exposure and eventual enhanced leukotriene production can cause respiratory hyperresponsiveness in association with eosinophilia. Mast cells also secrete chymase, which promotes eosinophil survival by dampening apoptosis cell programmes. Eosinophils themselves are able to extend their lifespan by releasing IL5 and CCL5 in auto/paracrine manner. Inflamed tissues propitiate eosinophil recruitment by releasing chemoattractant such as CCL5, CCL11, CCL24, and CCL26. TSLP has a major role in eosinophil recruitment into the respiratory tract. Eosinophils in turn jeopardize tissue integrity by disrupting the architecture of the extracellular matrix and by causing direct cellular damage through the release of specific granules content. Eosinophils are also able to interact with intravascular effectors of innate immunity such as platelets. Eosinophils contribute to platelet activation by releasing platelet activating factor (PAF) as well as MBP and EPO, while platelets affect eosinophil activation through the production of CCL5, CCL17, CXCL4, and IL1*β* and the engagement of P-selectin and CD40 with PSGL1 and CD40ligand, respectively. The reciprocal interactions between platelets and eosinophils favour the development of tissue inflammation and remodelling (especially at the level of the respiratory tract) and are possibly involved in the development of thrombosis. Activated eosinophils express tissue factor (TF) and are themselves able to promote thrombin generation. Under inflammatory conditions, eosinophils can also form extracellular traps of mitochondrial decondensed DNA, possibly contributing to the induction and maintenance of chronic inflammation.

Primary or secondary (see below) increases in the number of circulating eosinophils as well as inflammation-induced surges in the expression of eotaxins, IL5, or other chemoattractants (including complement anaphylatoxins C3a and C5a) cause the migration of inflammatory eosinophils towards nonphysiological homing tissues [17] (Figure 1). In these scenarios, T lymphocytes- and mast cell-mediated recruitment of eosinophils becomes more relevant. In addition, ILC-2, which play a major role in the physiological trafficking of eosinophils, are probably also coopted to divert eosinophils at sites of inflammation under pathological conditions [2, 18] (Figure 2). The heart is one of the preferential targets for eosinophil inflammation, as it is involved in up to one-third of patients with eosinophilic granulomatosis with polyangiitis (EGPA; see below) and up to half of the patients with (other) HES [19]. Furthermore, 0.5% of myocardial autopsies show signs of eosinophil infiltration irrespectively of the inciting cause [8]. The reason for a preferential homing of eosinophils in the myocardium under systemic inflammation is not clear. Impaired IFN*γ*-, Th17-, or NK-dependent responses have been claimed as potential favouring factors [5, 20]. Numerous other tissues such as the skin, the oesophageal mucosa, the biliary tract, and central or peripheral nerves and blood vessel walls might become pathological targets for eosinophil infiltration in a wide range of diseases. Upper and lower airways also constitute a preferential target for eosinophil spreading during inflammation. Furthermore, in this setting, consistent evidence has shown the presence of a clinical-pathogenic link between the course of eosinophilic inflammation in the nasal and sinus mucosa and in the lungs, leading to the concept of united airways disease (see below) [21]. Thymic stromal lymphopoietin (TSLP) is a crucial eosinophil chemoattractant to the respiratory tract [22].

Evidence from mice biology and, to a lesser degree, from studies involving human subjects suggests that housekeeping and inflammatory eosinophils constitute phenotypically and functionally distinct granulocyte subpopulations [13, 15].

### 1.3. Eosinophil Granules and Their Content

Intracellular organelles constitute the physical correlate of the functional specificity of eosinophils (Table 1). Eosinophil primary granules develop during the promyelocytic stage of differentiation and, unlike their neutrophil homonyms, are filled with a hydrophobic protein of the galectin family, called galectin-10. Galectin-10 accounts for the formation of Charcot-Leyden crystals (CLC) in tissues and biological fluids from patients with eosinophil inflammation and is thus also known as CLC protein [24, 25]. A recent study suggests a possible role of galectin-10 in T cell suppression [26].

The specific or crystalloid granules are larger than the primary granules and are armed with a vast array of cytotoxic basic proteins, which account for the characteristic acidophilic stain pattern of eosinophils. The crystal core of the specific granules is enriched with nonrenewable stores of major basic protein (MBP). MBP exerts cytotoxicity by interfering with the electrical homeostasis of the cell surface, which eventually leads to membrane permeability. In addition, MBP is also an important trigger for mast cell degranulation (Figure 2) [5]. Eosinophil-derived neurotoxin (EDN) and eosinophil cationic protein (ECP) are members of a highly polymorphic gene family of ribonucleases with a role in viral infections. EDN and ECP are both neurotoxic, whereas MBP has been shown to have neuroprotective effects [5, 25]. Eosinophil peroxidase (EPO), similarly to its neutrophil homologue myeloperoxidase, is involved in the generation of reactive oxygen species to digest extracellular pathogens [25]. EDN, ECP, and EPO concentrate at the periphery of MBP cores within the specific granules.

Lipid bodies constitute a third intracellular compartment, committed to the production of arachidonic acid derivatives such as leukotrienes and prostaglandins, which play a well-known role in the pathogenesis of airways inflammation and acute hypersensitivity reactions [24].

### 1.4. Core Granulocytic Features

Despite progressive functional specialization through the evolution, eosinophils retain several behavioural features from their granulocytic heritage. Such shared features have been first and better characterised in neutrophils due to their abundance in the circulating blood and at sites of inflammation but are progressively being recognised in eosinophils as well [27].

#### 1.4.1. Phagocytosis, Cell Killing, and Antigen Presentation

Similarly to neutrophils (although less effectively), eosinophils are able to phagocytose invading pathogens [28] and kill them intracellularly by delivering MBP and ECP to intracellular phagosomes [29]. This, in turn, paves the way to subsequent antigen presentation [14]. In addition, eosinophils are also endowed with extracellular killing mechanisms, which include releasing cytotoxins through degranulation, performing a respiratory burst through EPO [30] as well as extracellular DNA trapping [31]. Degranulation in eosinophils is tightly regulated. In most cases, small quanta of selected cytotoxins from the specific granules are released in the extracellular space (piecemeal degranulation), instead of a full-blown degranulation. Granule content release can also be delayed beyond the whole cell lifespan, as minefields of intact eosinophil granules, able to disassemble under inflammatory stimuli, have been observed [32].

#### 1.4.2. Eosinophil-Platelet Interactions and Thrombophilia

Besides playing a crucial role in physiological haemostasis, platelets contribute to the host defence as fundamental hubs of a complex network that involves the endothelium and circulating white blood cells. Platelets extend the ability of leukocytes to sense the presence of inflammatory stimuli and communicate with other cells either by direct cell-cell contact or by releasing bioactive compounds or microparticles. Aberrant platelet-neutrophil interactions have been consistently observed in a wide range of inflammatory diseases and constitute a potential target for therapeutic intervention [33, 34]. In the setting of eosinophil-driven inflammation, platelets can sense the presence of IgE-susceptible antigens through the expression of Fc*ε* receptors and assist the host response against parasites [35]. Eosinophils express P-Selectin Granulocytes Ligand 1 (PSGL1) on the cell surface, thus enabling the engagement of P-selectin on activated platelets [36] (Figure 2). Tripartite interactions among eosinophils, platelets, and the endothelium might also be favoured by CD40 ligand/CD40 interactions. CD40 ligand, in particular, can be expressed by eosinophils and platelets and bound by platelets and endothelial cells, prompting acute activation and long-term inflammatory responses [37, 38]. Soluble mediators such as eosinophil-derived platelet activating factor (PAF), MBP or EPO, and platelet-derived CCL5, CCL17 (also known as thymus and activation regulated cytokine, TARC), CXCL4 (also known as platelet factor 4 or PF-4), or IL1*β* can further enhance platelet-eosinophil interactions [35, 39, 40]. This, in turn, facilitates eosinophil extravasation towards inflamed tissues and prompts further platelet activation. Activated platelets affect chronic inflammation and long-term tissue remodelling through the release of mitogens [41] and are potentially endowed with an enhanced thrombogenic potential (although the evidence to this latter regard in the setting of eosinophilic inflammation is controversial) [35].

Besides interacting with platelets, activated leukocytes are themselves characterised by the ability to promote thrombosis by triggering the coagulation cascade. This can be achieved either by causing endothelial damage or by the expression of tissue factor (TF) [42, 43]. Eosinophils affect endothelial integrity by releasing EPO and constitute a relevant source of TF in hypereosinophilic syndromes [44–46]. Intriguingly, due to the extensive functional connections linking the coagulation cascade to the complement system and the kinins system, this latter feature may also influence a broader range of inflammatory responses in eosinophil-infiltrated tissues [47]. Genetic studies suggest that imbalances in the eosinophil cytokine network might affect vessel integrity and independently correlate with the risk of cardiovascular events [48].

#### 1.4.3. Eosinophil Extracellular Traps

Extracellular DNA traps formation (ETosis) is a recently described biological process that involves innate immune cells such as neutrophils, mast cells, and macrophages [49, 50]. During ETosis the nuclear components of the cell are extruded together with pattern recognition receptors and microbicidal moieties to generate organised grids of decondensed and biochemically edited chromatin that enhance microbial recognition and killing. ETosis has been extensively studied in neutrophils and the central role of neutrophil extracellular traps (NETs) in physiological host defence and in the induction of autoimmunity has been robustly proven [51]. More recently, a Swiss group reported that eosinophils are able to form extracellular traps (EETs) under inflammatory conditions as well [31] (Figure 2). A peculiar feature of EETosis is the presence of a highly immunogenic [52] mitochondrial, instead of nuclear, DNA within the extracellular traps. After their first description, EETs have been consistently detected in eosinophilic diseases such as atopic dermatitis, eosinophilic esophagitis [53], asthma [54], and, more recently, chronic rhinosinusitis with nasal polyps [55].

### 1.5. Pathogenic Interactions with Cells and Tissues

Eosinophils are part of a complex network of interactions that involves a large number of immunocompetent and nonimmunocompetent cells and tissues (Figure 2). Most relevant in this context is probably the axis between eosinophils and Th2 cells, which constitutes the core of the so-called type IVb delayed hypersensitivity reaction [56]. Th2 cells can stimulate eosinophils either directly, through the release of IL5 [7] or indirectly, by promoting a humoral adaptive response and in particular the production of IgE. Class E immunoglobulins can be recognised by eosinophils (through direct or platelet-assisted Fc*ε*R engagement) or activate mast cells during type I (immediate) hypersensitivity reactions. Mast cell derived compounds (such as prostaglandin D2, leukotrienes, CCL5, and IL5), in turn, stimulate eosinophils, which eventually cause tissue damage and are ultimately responsible for the persistence of the immune response following acute mast cell activation [57]. Activated mast cells also release chymase, which prevents eosinophils from undergoing apoptosis [58]. Eosinophils are able to maintain and exacerbate Th2 immune responses by providing plasma cells with survival factors (such as IL6 or A proliferation inducing ligand, APRIL) and by stimulating Th2 through IL25 [10, 59]. Interestingly, IL25 production can be regulated by the intestinal microflora, which in turn can affect the degree of eosinophil infiltration within the gut [60]. As previously discussed, ILC-2 determine the magnitude of eosinophil-mediated responses [2]. In addition, they provide a crucial link between the eosinophil/Th2 axis and inflamed tissues, since they readily respond to the release of inflammatory stimuli such as IL25, IL33, or TSLP from epithelial cells and stimulate Th2 through the release of IL4 [3].

After recruitment into inflamed tissues, eosinophils cause tissue damage by generating oxidative stress through EPO, by disrupting the architectural organisation of the extracellular matrix, by prompting cell cytotoxicity through granule proteins such as ECP or through antibody-dependent cell cytotoxicity [61]. The release of mitogens (either direct or platelet-mediated) has a central role in long-term tissue remodelling, especially in chronic diseases such as asthma [62]. In addition, eosinophil-induced thrombosis can result in the loss of functional tissue by means of ischemia [46]. Fibrosis constitutes the final stage of inflammation-induced maladaptive responses to tissue injury. Eosinophils can promote fibrosis directly, by releasing transforming growth factor *β* (TGF-*β*), IL4, and IL13 [63, 64], or indirectly, by stimulating tissue-residing epithelial cells through MBP or EPO to express profibrotic mediators [65]. Upstream eosinophil activation, ILC-2 can also promote tissue fibrosis by secreting IL13 [66].

### 1.6. Pharmacological Modulation of Eosinophil Biology

#### 1.6.1. Drugs Exerting a Cytotoxic Effect on Eosinophils

Glucocorticoids have historically been the first and most effective drugs employed to dampen eosinophil-mediated damage in neoplastic and nonneoplastic conditions. Similarly to the pleiotropic effects on other leukocytes, glucocorticoids cause eosinophil apoptosis and inhibit the release of cytokines involved in eosinophil survival [4]. Hydroxyurea can also be employed as a first-line treatment in noninflammatory HES, also in combination with corticosteroids. Interferon alpha is usually considered a second choice due to the high rate of side effects. The expression of CD52 on the surface of eosinophils supports the use of the monoclonal antibody alemtuzumab beyond its conventional employment for severe T cell-mediated neoplasms or inflammatory diseases [67]. Imatinib and other tyrosine kinase inhibitors (TKI) are highly effective in hypereosinophilia due to clonal myeloid diseases with known chromosomal rearrangements (see below), while they should not affect idiopathic HES (iHES) or HEUS. Nonetheless, recent studies using next-generation-sequencing (NGS) showed that a subset of patients provisionally diagnosed with iHES or HEUS harbour point mutations that prompt clonal myeloid haematopoiesis (see also below) [68]. These studies suggest that TKI might also play a therapeutic role at least in some patients with apparent iHES/HEUS [69–72].

Conventional antiasthmatic drugs such as theophylline and antileukotrienes have been shown to promote eosinophil death besides their anti-inflammatory effects, whereas beta2-agonists favour eosinophil survival [73]. Several novel potential strategies based upon the promotion of eosinophil apoptosis are under development and include targeting surface molecules such as sialic acid-binding immunoglobulin-like lectin 8 (Siglec8, which, however, is expressed by both inflammatory and regulatory eosinophils [13]), factors involved in the control of the cell cycle and DNA rearrangement, and regulators of the intracellular ionic balance [4, 73]. In particular, levosimendan and its analogues are calcium sensitisers employed as inotropes in severe heart failure and exert proapoptotic effects on eosinophils* in vitro *[74]. Accordingly, they might find a role in eosinophil-mediated diseases, especially in eosinophilic myocarditis, although no clinical evidence has been so far published in this regard.

#### 1.6.2. Other Cytotoxic Drugs

Conventional immune suppressants, such as cyclophosphamide, are employed to induce remission through the control of T and B cell activity in neoplastic and inflammatory diseases [67, 75]. Rituximab, an anti-CD20 monoclonal antibody, has an established role in definite B cell-mediated diseases but has also relevant upstream effects on the whole Th2-centred network [76–78]. Accordingly, its efficacy has been proven also in some eosinophil-related diseases such as EGPA [75]. Other immune suppressants such as mofetil mycophenolate, methotrexate, or azathioprine are employed as steroid sparing agents mainly in inflammatory diseases [75, 79].

#### 1.6.3. Anticytokine Drugs

In recent years, novel classes of drugs targeting specific cytokines in the eosinophil signalling network have been introduced. These agents have been designed to dampen the effects of eosinophilia on target organs, rather than causing a general immune suppression. Accordingly, in contrast to the past, their clinical development occurred first in immunorheumatological rather than haematological settings. The anti-IL5 monoclonal antibodies mepolizumab and reslizumab were able to improve asthma disease course in randomised clinical trials (see below) [7]. Furthermore, there is evidence of efficacy of mepolizumab in inducing disease remission in selected EGPA subsets and disease stabilisation in patients with HES [67, 80]. Similarly, benralizumab, an anti IL5-R alpha chain antibody, showed promise in eosinophilic asthma and might also potentially be applied to other clinical settings, due to its additional ability to deplete eosinophils through antibody-dependent cytotoxicity [4, 7]. TPI ASM8 is small oligonucleotide, designed for inhaled administration, which exploits RNA interference to dampen the expression of CCR3 and of the shared IL3-R, GM-CSF-R, and IL5-R beta chain. Preliminary clinical data suggests its efficacy in the control of eosinophil inflammation [81]. GW766994 a selective CCR3 competitive antagonist has recently been tested in patients with asthma and sputum eosinophilia (NCT01160224). The results of the trial have not yet been published. Drugs targeting signalling pathways characterised by redundancy, such as CCL11, IL4, and IL13, have shown limited clinical efficacy, whereas others, such as those targeting IL33, seem more promising [4].

#### 1.6.4. Other Current or Future Therapeutic Strategies

As mast cells are preferential partners in the signalling interchanges between eosinophils and other inflammatory cells, inhibitors of mast cells are expected to affect eosinophil biology. Indeed, the anti-IgE antibody omalizumab has disproportionately positive effects in symptoms control in asthma as well as in nasal polyposis, possibly because of a downstream effect on the recruitment and activation of eosinophils [82]. Novel antimast cell therapies include interfering with prostaglandin D2 or histamine signalling pathways [4].

Inhibition of cell migration into inflamed tissues has revealed a promising strategy in different inflammatory diseases [83, 84] and may be applied to disorders characterised by eosinophil infiltration. However, potential drawbacks can also arise, as a result of eosinophil intravascular pathogenicity [4].

## 2. Eosinophils in Infectious Diseases

### 2.1. Parasitic Infections

Eosinophils have classically been associated with host defence against parasitic infections, particularly caused by helminths, due to the documented* in vitro* ability of larval killing [85, 86]. However, more recent studies highlighted a dual role of eosinophils in parasitic infections, as these cells exert a protective activity alternatively for the host or for the worm. Recent reviews analysed in detail the mechanisms involved in eosinophils-related host-pathogen interaction [87–89]. Experimental approaches employing eosinophil-ablated mice allowed a better understanding of this bivalent role [87], although with some intrinsic limitations that do not permit to draw definite conclusions regarding the* in vivo* contribution of eosinophils to defence against helminth infections.

In animal models, eosinophils were shown to accumulate around dying* Taenia solium* parasites [90–92], and an eosinophilic response in humans affected by neurocysticercosis is evident in the cerebrospinal fluid [93]. However, the bystander effect of the inflammatory response may be detrimental to the nervous tissue, and in an eosinophil-ablated mouse model a higher parasite burden was associated with less severe disease, enhanced survival, and reduced tissue damage and neuroinflammation [94].

A similar finding was evident in an eosinophil-ablated mouse model infected with* Schistosoma mansoni, *where eosinophils had no impact on worm burden, egg deposition, and liver granulomas number, size or associated fibrosis and hepatocellular damage [95]. In fact, in IL5-knockout mice infected with* Schistosoma mansoni*, granulomata were completely devoid of eosinophils and were shown to have a smaller size. In addition, the animals showed reduced liver fibrosis [63].

Eosinophils were shown to be able to kill larval* Strongyloides stercoralis* [96] and to act as antigen-presenting cells stimulating T cell proliferation, Th2 cytokine production, and antibody production by B cells [97]. However, in eosinophil-depleted mice the eosinophil response was shown to be dispensable during primary infection, as both neutrophils (through myeloperoxidase-mediated killing) and eosinophils (through MBP-mediated killing) were able to act as effector cells in the primary response against* Strongyloides stercoralis* [98].

The contrasting role of eosinophils during primary or secondary parasitic infection is exemplified by Trichinosis and filarial infections. In eosinophils-ablated mice,* Trichinella spiralis* larvae survival is reduced and parasite death correlated with enhanced IFN*γ* and decreased IL4 production [99]. Moreover, recent studies showed that eosinophils, along with IL4, support larval growth by suppressing local inflammation and IFN-driven responses [100] and produce IL10, which promotes expansion of CD4 T cell and dendritic cells. These latter cell subsets, in turn, are able to reduce NO production by inhibiting inducible NO synthase expression, finally limiting larval killing [101]. However, during secondary infection eosinophils exert a protective effect by limiting muscle larvae burden, probably by an antibody-mediated binding mechanism [102]. On the other hand, In* Brugia malayi* primary infection, eosinophils are required for the innate clearance of microfilariae through a CCL11-dependent mechanism [103], while eosinophils do not appear to be required in the control of secondary infection [104]. Interestingly, eosinophil granule proteins are not essential for protection during primary infection [105]. Nevertheless, in infections due to* Onchocerca volvulus*, eosinophils appear to be required for protective immunity [106].

Among the nematodes, members of the Anisakidae family (the most common being* Anisakis simplex*) are the aetiological agents of gastric, ectopic, and intestinal anisakidosis. The infection may become chronic and prompt the formation of granulomata, which in turn may require surgical intervention [107] or even be misdiagnosed as neoplasms (often referred to as “vanishing tumours,” due to their frequent, spontaneous tendency to disappear [108]). Interestingly, the spectrum of diseases related to* Anisakis *spp. and similar microorganisms constitute also a paradigm of the pathogenic links between allergy and parasitic infections. In fact, allergic sensitisation to* Anisakidae *is frequent, especially in countries where raw fish/seafood consuming habits are diffused, such as Japan, Korea, Spain, or Italy [109, 110], due to the high prevalence of these worms in commercially relevant species [111]. In addition,* Anisakis* larvae can elicit a strong Th2-driven inflammatory response, characterised by prominent eosinophil activation. Specifically,* Anisakis* larvae release a panel of toxins that act as potent chemoattractants for eosinophils [112], which in turn exploit anti-*Anisakis* antibodies to adhere to the* Anisakis* epicuticle and to progress into further cell activation stages towards the release cytotoxic factors such as MBP and ECP [113, 114]. Unfortunately, this has no effect on the nematode but may contribute to host tissue damage [114]. Curiously,* Anisakis* toxins are also endowed with a potential cross-reactivity with wasp venom allergens [115]. Hypersensitivity reactions due to Anisakis exposure (including life-threatening anaphylaxis [116]) are thus not infrequent and may coexist with the complications of acute infection. In particular, an overlapping condition encompassing allergic and infectious features was defined by some authors as “gastroallergic anisakiasis” [117, 118]. Indeed, it is still not clear whether live* Anisakis* larvae are required for allergic reactions, or if proteins of dead larvae may also act as triggers, although it appears that living larvae are necessary for both initial sensitisation and subsequent allergic reactions. Nevertheless, cases of reaction to proteins of dead larvae have been described [110]. Interestingly, the Th1/Th2 balance of the immunological response against* Anisakis* was shown to be associated with the clinical manifestations of the disease: in patients with a Th1-prevalent response gastrointestinal symptoms predominated, while a response biased towards Th2 was more frequently found in patients with generalised allergic symptoms [119].

From a clinical point of view, eosinophilia may be a diagnostic clue for a helminth infection, especially if accompanied by fever and other manifestations related to the anatomic site of infection. A careful medical history, with particular focus on risk factors and exposure to endemic pathogens (i.e., travel history), and physical examination usually guide the differential diagnosis, leading to specific diagnostic tests to confirm the aetiology. In general, eosinophilia in helminth infection is more frequent and more pronounced in acute-early infection. On the other hand, some pathogens, such as* Strongyloides *spp.,* Echinococcus *spp.,* Schistosoma *spp., and the filarial worms may present with eosinophilia even decades after primary infection. Of note, systemic eosinophilia in patients with anisakidosis is described in less than 30% of cases [116]. Other protozoa are less likely to cause eosinophilia, with the notable exception of* Sarcocystis *spp. [120] and* Cystoisospora *spp. [121]. A detailed review of eosinophilia differential diagnosis in infectious diseases was recently published by O'Connell and Nutman [122].

### 2.2. Bacterial Infections

As discussed above, eosinophils are able to perform bacterial killing through several intra- and extracellular mechanisms, which interestingly may vary according to the involved pathogens [30, 88]. Specifically, intracellular killing mechanisms were demonstrated for* Staphylococcus aureus*,* Escherichia coli,* and* Listeria monocytogenes* [88]. Several studies evaluated the behaviour of eosinophils during acute bacterial infections, where eosinopenia has been shown to be a common feature [17]. During bacteremia, there is an inverse relationship between bacterial load and peripheral blood eosinophils [123] and eosinopenia was shown to be a reliable marker of bacterial aetiology in patients admitted with sepsis to the intensive care unit [124–126]. Finally, low eosinophil count was shown to be a risk factor for persistent diarrhoea or death and recurrent disease in patients with* Clostridium difficile* infection [127]. Interestingly, a recent report showed that IL25-related eosinophilia might reduce the severity of* Clostridium difficile* infection, possibly due to the regulation of the immune response preventing disruption of the intestinal barrier [60].

### 2.3. Fungal Infections

*In vitro* studies showed that eosinophils challenged with* Alternaria alternata* (a common environmental fungus) can be activated by recognition of *β*-glucan (a common component of fungal cell wall) through CD11b or after cleavage of protease-activated receptor 2 (PAR-2) by* Alternaria*'s proteases [128, 129]. Eosinophils respond to these stimuli by effectively releasing proinflammatory and cytotoxic granule proteins (such as EDN or MBP) and several chemokines (namely, CCL2, CCL3, and IL8) [128].

Eosinophil behaviour in the spectrum of diseases caused by* Aspergillus *spp. constitutes an additional example of the pathophysiological bonds between host defence and allergy. The Aspergillus cell wall component chitin was shown to promote lung eosinophil recruitment and a Th2-skewed immune response, although a specific receptor binding chitin has yet to be characterised [130]. Eosinophils were shown to be involved in the immune response against the infection by contributing to the clearance of* Aspergillus* from the lung, as eosinophil-deficient mice demonstrated impaired clearance and increased fungal germination. Moreover, a potent killing activity of eosinophils against* Aspergillus* was shown to take place without the need for direct cell contact, suggesting a fundamental role of proinflammatory cytokines and chemokines released by degranulation [131]. A recent study provides evidence of a dual behaviour of eosinophils after challenge with* Aspergillus fumigatus* [132]. While the conidial killing ability of eosinophils and the hypersusceptibility to* Aspergillus* infection of eosinophil-ablated mice were confirmed, eosinophils were also shown to be a prominent source of IL-23 and IL-17, which might play a crucial, detrimental role in the induction and maintenance of inflammation in allergic aspergillosis (see also below).

The role of eosinophils in other fungal infections such as cryptococcosis has been explored. Eosinophils are able to phagocytose* Cryptococcus neoformans* and to present its antigens to immunocompetent cells. In addition, exposure to* Cryptococcus* prompts eosinophils to release IL12, IFN*γ*, and TNF [133]. On the other hand, eosinophils might have an immunoregulatory role in pulmonary cryptococcosis due to the production of IL4, which promotes a Th2-driven inflammatory response that favours lung damage and pathogen dissemination [134]. Clinically, eosinophilia, albeit rare, may also be a clue for the diagnosis of disseminated cryptococcosis [135].

Peripheral blood eosinophilia can also occur in other infections due to fungi, such as* Coccidioides immitis* [136]* Paracoccidioides brasiliensis* [137] and* Histoplasma capsulatum* [138], but the role of eosinophils in these settings remains to be fully elucidated.

### 2.4. Viral Infections

Since eosinophils are key players in allergic and granulomatous diseases, their role in the immunopathogenesis of viral infections has specifically been evaluated in the field of respiratory infections.

In infections due to respiratory syncytial virus (RSV), especially in infants, eosinophils are recruited in the lower airway epithelium [139]. RSV itself can activate eosinophils [140], which are in turn able to promote virus clearance and reduce airway dysfunction through direct mechanisms, such as production of ribonucleases, and indirect mechanisms mediated by the production of several cytokines promoting host defence (e.g., IFN-*β*) [141]. Eosinophils are also involved in host response to influenza viruses. Recent studies showed that, after challenge with influenza A virus, they are able to undergo piecemeal degranulation, upregulate antigen presentation markers, and enhance CD8+ T cell response [142]. This mechanism is particularly relevant in light of several retrospective studies that showed that, during the 2009 influenza pandemic, patients with asthma had a higher risk of being hospitalised, but a lower risk of complications or death [143, 144], thus highlighting a possible role of eosinophils in the immune response against influenza virus* in vivo*. An antiviral activity of eosinophils has also been shown for other respiratory viruses, such as parainfluenza virus. In this case, different mechanisms such as producing nitric oxide and upregulating TLR7 as well as acting as cellular decoys to limit viral replication have been described. By contrast, a direct effect of RNases and other excreted proteins has not been observed [145]. Evidence suggests that eosinophils contribute also to the host response against rhinoviruses by inducing a T cell virus-specific response [146].

Besides respiratory viruses, eosinophils play a role in other viral infections. Eosinophilia is a frequent finding in patients with HIV infection progressing to full-blown AIDS, even in the absence of other triggers such as parasitic infections or allergic condition [147, 148]. Indeed, human eosinophils express CD4 and CXCR4 [149, 150] and are susceptible to infection by CXCR4-tropic HIV-1, according to evidence* in vitro* [151–153]. Interestingly, although eosinophils also express CCR5 [89],* in vitro* studies showed that only CXCR4-tropic HIV strains can give rise to productive infection [154] and some authors speculate that the inability of CCR5-tropic viruses to actively infect eosinophils may be due to the necessity of higher levels of CCR5-expression [155], as shown for CD4+ T cells [156]. Nevertheless, the* in vivo* immunopathogenic mechanisms of eosinophil infection by HIV have yet to be fully elucidated. In chronic patients with disease progression, a change in the immune response from a Th1-predominant to a Th2-predominant phenotype is evident [156], and this shift in cytokine production towards a Th2 pattern further impairs CD8+ T cell response of the host against HIV [157]. On the other hand, an increased production of IL5 due to this unbalanced Th2 response might explain, at least in part, the increased eosinophil count seen in patients progressing to AIDS [155].

## 3. Eosinophils in Haematological Disorders and Cancer

### 3.1. Eosinophilia in Clonal Disorders

Eosinophilia can be present in both myeloid (chronic myeloid leukaemia, acute myeloid leukaemia, systemic mastocytosis, and myelodysplastic/myeloproliferative diseases) and lymphoid (Hodgkin's lymphoma, T cell non-Hodgkin lymphomas) malignancies (Table 2). Oncohaematological disorders should be suspected when infective or immunological causes of persistent hypereosinophilia have been excluded.

Different pathogenic mechanisms can underlie eosinophilia in haematological clonal disorders. In myeloid malignancies, a genetic lesion (chromosomic rearrangements, point mutations) in the hematopoietic stem cell, mainly involving tyrosine kinase (TK) genes, results in dysregulation of cell signalling/proliferation, with direct expansion of the eosinophil compartment [158]. In lymphoproliferative diseases or lymphoid leukaemia, one or more genetic lesions result in lymphoid or blast expansion, and eosinophilia can be present as part of a paraneoplastic microenvironment. Patients with lymphocyte-variant primary eosinophilia have no overt haematological malignancy, but their haematopoiesis is characterised by occult expansion of immunophenotypically aberrant T lymphocytes, which produce cytokines such as IL5 (Figure 2) [159–161].

Molecular biology dramatically changed the definition of primary eosinophilia. Evidence about genetic mutations/rearrangements causing eosinophil expansion is maturing with the recognition of a new specific World Health Organization category, named “Myeloid/lymphoid neoplasms with eosinophilia and rearrangement of* PDGFRA*,* PDGFRB*, or* FGFR1*, or with* PCM1-JAK2*” [162]. In the absence of infective and/or immune-rheumatologic causes of eosinophilia, examination of the blood smear and blood tests (looking for circulating blasts, dysplastic cells, monocytosis, and tryptase elevation) can confirm the suspicion of a clonal disease. Further diagnostic work-up entails screening on peripheral blood for the most common genetic lesions involved in clonal eosinophilia:* BCR-ABL, JAK2V617F, FIP1L1-PDGFRA, ETV6-PDGFRB* gene fusions,* KITD816V* mutation, and T cell receptor (TCR) clonal rearrangement. Bone marrow aspiration and biopsy are needed to define the diagnosis through morphological examination and cytogenetics. Fluorescence in situ hybridisation (FISH) analysis is used to detect the presence of the cytogenetically occult rearrangement (mostly deletions) resulting in gene fusions, as a proof of clonality [163]. Molecular genetics has also had a deep impact on the therapeutic scenario, paving the way to the success of TKI [69–72]. As previously introduced, thanks to the availability of NGS techniques, several novel mutations are expected to be found in patients with iHES or HEUS, based on the observations made in large cohort studies [164]. For example, Schwaab and colleagues studied 426 patients with HEUS enrolled in the German Registry on Disorders of Eosinophils and Mast cells and found a prevalence of 12%, 4%, and 3% for* FIP1L1-PDGFRA, KITD816V,* and* JAK2V617F* lesions, respectively. Additional mutations (mainly in* TET2* and* SRSF2* genes) were also identified in a subset of patients with* KITD816V* positivity. Most importantly, the authors showed that the molecular profile correlated with the clinical outcome and could support the use of highly effective targeted therapies [165]. In another study, Wang et al. employed NGS analysis to assess the presence of cryptic mutations in bone marrow samples from 51 patients with iHES and found a 28% prevalence of single or multiple mutations in* ASXL1, TET2, EZH2, SETBP1, CBL,* and* NOTCH1* genes. Consistently with the observations of Schwaab et al., the authors reported a molecular/prognostic correlation. In particular, patients with iHES and NGS-positive genetic lesions had a survival profile comparable to that of patients with chronic eosinophilic leukaemia (CEL) not otherwise specified [166].

#### 3.1.1. Eosinophils in Myeloid Neoplasms

Philadelphia-positive BCR-ABL^+^ chronic myeloid leukaemia (CML) classically presents peripheral neutrophilia, basophilia, and eosinophilia; in rare cases the disease presents with prominent hypereosinophilia, as the eosinophilic variant of CML (eoCML). Abnormal expansion of eosinophil compartment in blood at time of CML diagnosis was traditionally recognised as an unfavourable factor, representing one of the elements of a classic prognostic score of CML (Hasford score) [167]. As in CML, eosinophilic myeloproliferative neoplasms (eoMPN) with FIP1L1-*PDGFRA, *ETV6-*PDGFRB, FGFR1,* or* PCM1-JAK2 *rearrangements are characterised by gene fusions caused by chromosomic translocations. These, in turn, prompt constitutive activation of the TK domain, causing direct clonal expansion of eosinophils [67, 168]. In cases where* JAK2, BCR-ABL, PDGFRA, PDGFRB, and KIT* mutation are not found, CEL should still be ruled out before confirming a diagnosis of iHES or HEUS. CEL is defined by the presence of hypereosinophilia with clonal cytogenetic or molecular genetic abnormality, or when blast cells are more than 2% in the peripheral blood or more than 5% (but less than 20%) in the bone marrow [67]. Both eoCML and Philadelphia-negative eoMPN can present with eosinophil-related organ damage including perivascular tissue fibrosis and vasculitis [169].

Systemic mastocytosis is characterised by clonal expansion of mast cells, in most cases due to cKIT mutations [170], and by an extensive release of their large array of proinflammatory mediators, which take part in eosinophil signalling network and sustain a positive feedback loop (Figure 2). Accordingly, up to 28% of patients with systemic mastocytosis have peripheral (nonclonal) eosinophilia, and bone marrow eosinophilia is frequently detectable, even in cases without significant increase of peripheral eosinophil count. Nonetheless, the clinical phenotype is largely driven by mast cell activation and ranges from absence of symptoms to severe recurrent anaphylaxis [171].

Eosinophil count is also increased in some cases of acute leukaemia (blast count more than 20% in bone marrow), in particular acute myelomonocytic leukaemia (FAB M4) or AML with inversion of chromosome 16 (2016 WHO category). Although eosinophilia has limited pathogenic relevance in these diseases, it may affect the therapeutic outcome in M4-AML patients [172].

Finally, eosinophilia is a typical accompanying feature of Langerhans cell histiocytosis.

#### 3.1.2. Eosinophils in Nonmyeloid Haematological Disorders

Both chronic and acute lymphoid clonal disorders can associate with eosinophilia. The “Lymphocytic variant of HES” (L-HES) constitutes a separate nosologic category. L-HES is characterised by clonally expanded circulating mature T cells, extensive release of IL5 with eventual eosinophil recruitment (Figure 2), and low risk of malignant transformation [173–177]. Abnormal T cell expansion in L-HES may be characterised by a lack of expression of both CD4 and CD8 antigens (CD3+ CD4− CD8− cells) or by CD3 negativity (CD3− CD4+ cells). Additional surface abnormalities include an aberrantly elevated CD5 expression, loss of CD7, and/or expression of CD27 [67]. Molecular biology analysis is used to demonstrate TCR clonality. From a pathophysiological perspective, the disease resembles a nonneoplastic inflammatory disorder (see below). Accordingly, the clinical phenotype is dominated by skin and soft tissue inflammation, although cardiac, pulmonary or constitutional manifestations may also develop.

In other lymphoid malignancies, eosinophils can contribute to the neoplasia-associated microenvironment. Peripheral blood increases in eosinophil count are commonly described in Hodgkin's Lymphoma (HL), in which Reed-Steinberg cancerous cell is largely surrounded and supported by host hematopoietic cells at sites of tissue infiltration. Eosinophilia is present in 15% of HL at diagnosis. Only rarely HE criteria are also met [178, 179]. Furthermore, eosinophilia can be a feature of other chronic lymphoid diseases as well as of acute lymphoblastic leukaemia [180–183], where it can associate with organ damage and affect prognosis. An overall treatment response usually correlates with remission of eosinophilia.

#### 3.1.3. Eosinophils in Graft-versus-Host Disease

Host tolerance after allogeneic hematopoietic stem cell transplantation not only involves non-self-recognising donor lymphocytes, but also innate immune cells. In fact, acute GvHD is frequently observed at time of granulocyte engraftment or soon thereafter [184–186]. Eosinophil count has been largely studied as a risk factor and predictor of severity for both chronic and acute GvHD, but the real pathogenic role of eosinophils in GvHD is controversial. Some authors showed that eosinophil expansion in chronic GvHD can correlate with better prognosis, hypothesizing eosinophilia as a surrogate for Th1/Th2 imbalance in favour of Th2-type and B cell-mediated alloreactivity, which, in turn, could result in less severe, mainly mucocutaneous forms of chronic GvHD [187].

### 3.2. Eosinophilia in Solid Tumours

Myeloid cells play a fundamental role in the inflammatory response against tumours and in the development of the peritumour microenvironment [188]. Eosinophils constitute a significant fraction of the leukocyte infiltrate surrounding different cancer histotypes. Their role and clinical relevance in this setting are unclear, as conflicting results have been reported [16, 189]. Eosinophils are thought to provide a stereotyped response towards necrosis (a hallmark of cancer biology) either favouring an antitumour inflammatory response or a protumour misrepair response with enhanced angiogenesis and release of growth factors, depending on the surrounding stimuli [57, 189, 190]. Numerical increases in the number of circulating eosinophils may also constitute paraneoplastic epiphenomena. Furthermore, activated eosinophils might contribute to thrombophilia in patients with cancer [191, 192].

## 4. Eosinophils in Immune-Mediated Diseases

Deranged eosinophil function might occur as a result of a complex combination of genetic and environmental factors. Unbalanced Th2-responses often associate with eosinophilia and/or eosinophil-mediated tissue damage. Not surprisingly, eosinophilia is a hallmark of several allergic diseases and of EGPA, which we will extensively discuss in this section. In addition, it should be mentioned that recent evidence suggests a prominent role of eosinophils in Devic's syndrome (neuromyelitis optica) and primary biliary cirrhosis [5]. Finally, eosinophilia can also be frequently observed in a wide range of other immune-mediated diseases such as pemphigoid, bullous pemphigoid, systemic sclerosis, sarcoidosis, or IgG4-related disease [193, 194].

### 4.1. The Role of Eosinophils in Selected Skin Diseases

The skin is devoid of eosinophils under physiological conditions [195]. Several dermatological diseases of various aetiology show both peripheral and/or tissue eosinophilia. Among allergic diseases, urticaria, atopic dermatitis, and delayed drug hypersensitivity reactions are the main conditions associated with increased tissue and/or peripheral eosinophils. Other dermatological diseases that have to be considered in the differential diagnosis include psoriasis, bullous diseases, palmoplantar keratoderma, and malignancy [5, 196].

#### 4.1.1. Atopic Dermatitis

Atopic dermatitis (AD) is an inflammatory disease of the skin, characterised by epithelial dysfunction (either congenital or maintained by inflammation itself) with parakeratosis and by a significant expansion of the Th22 compartment [197, 198]. The defective barrier function of the skin in AD facilitates antigen penetration and exposure to the immune system, thus paving the way to enhanced, aberrant humoral, and/or cellular immune responses. Accordingly, AD is associated with other autoimmune diseases encompassing skin manifestations and with respiratory and food allergies [199]. IgE are usually elevated in AD and can recognise self- or non-self-antigens. Nonetheless, their pathogenic role in AD is still only partially understood [198, 200, 201]. It has been proposed to define two different AD endotypes, the former being characterised by a prominent Th2 profile, as opposed to a non-Th2 profile featuring a combination of Th1- and Th17-driven inflammation [202–204]. Cytokine activation patterns are different in patients with extrinsic (allergic) and intrinsic (nonallergic) AD, but both subtypes show similar Th2 activation regardless of IgE status. The effectiveness of dupilumab, an anti IL4 and IL13 monoclonal antibody, in AD indicates that, in contrast to other diseases such as asthma [4], these cytokines might play a nonredundant role in the disease pathogenesis. Eosinophil infiltration and blood eosinophilia constitute additional hallmarks of AD. Blood eosinophilia has been shown to correlate with disease severity. On the other hand, the role of tissue eosinophils is less clear, since they can either contribute to damage or assist host defence against superimposed infections and promote the autoregulation of the immune response [200]. Due to our incomplete understanding of the disease, current therapeutic strategies are nonspecific and comprise the use of emollients, topical/oral immunosuppressant therapy (corticosteroids, tacrolimus/pimecrolimus, and cyclosporine). Targeted therapies show promise for the next future. Most robust evidence in this regard has been acquired with dupilumab, whereas data from other biologics such as omalizumab are scanty [205].

#### 4.1.2. Chronic Spontaneous Urticaria

Chronic spontaneous urticaria (CSU) is a heterogeneous mast cell-related disease, characterised by recurrent flares of wheals and/or angioedema lasting for >6 weeks, generally in the absence of clear offending triggers. Histological evidence suggests that eosinophils are abundant, along with mast cells and expanded microvasculature, at sites of skin lesions and even of healthy skin in patients with CSU. These data might indicate that eosinophils are involved in a skin priming process dominated by vessel remodelling, which in turn facilitates subsequent wheals formation [206]. In addition, eosinophils can trigger the typical acute changes in vascular permeability by interfering with the network between the coagulation cascade, the complement system, and the kinin system (see also above and Figure 2) [207]. From a clinical perspective, the paradigmatic link between eosinophilic inflammation and antiparasitic response is underlined by the disproportionate susceptibility of patients with CSU to* Toxocara* seropositivity and* Anisakis simplex* sensitisation [208].

#### 4.1.3. Gleich Syndrome

In patients with recurrent angioedema and eosinophilia, Gleich syndrome should be suspected. It consists in a rare and benign disease, characterised by recurrent episodes of angioedema, urticaria, fever, significant increase of body weight (15–20%), and a remarkable elevation of eosinophils and IgM. Each cycle lasts approximately 25–30 days. Gleich syndrome has a benign natural history, as it does not involve internal organ and recovers spontaneously or with a short course of oral corticosteroids. Recently, an aberrant CD3−CD4+ T cell population with a clonal pattern of expression of the TCR has been consistently demonstrated in patients with Gleich syndrome [209]. Nonetheless, the aetiology of the disease and the factors supporting its cycling pattern remain unknown. The diagnosis should be made by exclusion of underlying disorders causing oedema (such as heart, kidney, and liver diseases) and/or hypereosinophilia (such as allergy, parasites infections, collagen diseases, or other haematologic diseases) [210].

### 4.2. Drug Hypersensitivity Reactions with Eosinophilia

#### 4.2.1. Nonimmediate or Cell-Mediated Drug Hypersensitivity Reactions

Delayed or type IV drug hypersensitivity reactions (DHR) include immune-mediated reactions that occur more than one hour after the drug administration. Reactions severity range from self-limited maculopapular rashes that recover after drug suspension to toxic epidermonecrolysis, a life-threatening reaction with resulting organ damage and a high rate of mortality [211, 212]. Delayed DHR (dDHR) are often accompanied by peripheral/tissue hypereosinophilia, thus qualifying themselves as type IVb reactions (see above). The main clinical entities included in this subgroup of dDHR are isolated peripheral hypereosinophilia, maculopapular rash, and drug-reaction with eosinophilia and systemic symptoms (DRESS).

#### 4.2.2. Isolated Peripheral Hypereosinophilia

Many drugs can induce a benign hypereosinophilia, but the exact underlying pathogenic mechanisms are far from being understood. Eosinophilia can constitute an expected side effect of certain cytokine therapies (e.g., IL2, GM-CSF) which cause an IL5 surge, whereas in other cases it represents a DHR. In particular, penicillins, sulfonamides, aromatic anticonvulsants (including phenobarbital and carbamazepine), and heparin and TNF antagonists have been described to cause isolated hypereosinophilia [213, 214].

#### 4.2.3. Maculopapular Exanthema

Maculopapular exanthema is the most frequent manifestation of dDHR. Hypereosinophilia occurs in approximately 50% of severe cases [215]. In such cases, viral aetiologies should be excluded, especially in children [212].

#### 4.2.4. DRESS or Drug-Induced Hypersensitivity Syndrome (DIHS)

Drug-reaction with eosinophilia and systemic symptoms (DRESS) is a peculiar DHR characterised by a maculopapular rash accompanied by constitutional symptoms and multiorgan failure (Table 3). In particular, the liver and the kidneys are frequently involved. Similarly to other DHR, the pathogenesis of DRESS encompasses an abnormal adaptive T cell response. Thus, not surprisingly, reactions to specific drugs segregate with specific HLA subsets and are affected by the genetic background. In addition, there is evidence that the risk of developing DRESS after exposure to a given drug is ethnicity-specific and that ethnicity can also affect the clinical course of the disease [216, 217]. Multiple drug classes can induce DRESS. However, antiepileptic drugs and antibiotics are more frequently implicated [217–221]. According to the current pathogenic paradigm, drug-specific CD4^+^ T cells activate and promote a CD8^+^-mediated immune response towards different tissues. The reactivation of herpesviruses (in particular HHV-6) has also been claimed as a major driver of the deranged T cell response [222]. Eosinophils proliferate and are recruited at sites of tissue injury following the release of IL5, CCL11, and CCL17 [215, 223]. The onset of DRESS usually occurs 2–8 weeks after starting the culprit drug, which implies an even higher temporal delay, when compared to other dDHR. In addition, persistence or evolution of the rash and of the organ failure despite drug discontinuation may occur, possibly as the consequence of concomitant viral reactivation [224]. The diagnostic work-up of DRESS includes patch tests and intradermal skin test with delayed reading, when clinical history is not able to identify the culprit drug. These tests should be performed at least 6 months after the resolution of the reaction.

### 4.3. Gastrointestinal Manifestations of Eosinophilic Inflammation

#### 4.3.1. Eosinophilic Oesophagitis

Eosinophilic oesophagitis (EoE) is as a chronic inflammatory condition of the oesophagus characterised by an eosinophilic infiltration with ≥15 eosinophils per high-power field (HPF). The currently accepted pathogenic paradigm in EoE suggests that antigens, possibly facilitated by alterations in the oesophageal epithelial barrier, are taken up by antigen-presenting cells, which promote a Th2 polarisation. TSLP plays a key role in this setting, by enhancing the interactions between dendritic cells and T cells [225]. Histologically, oesophageal biopsies from patients with EoE may have eosinophil surface layering, eosinophilic microabscesses, and increased levels of dendritic cells and degranulating mast cells. Oesophageal inflammation is accompanied by basal layer hyperplasia and dilated intracellular spaces. Later stages are characterised by fibrosis of the lamina propria, which accounts for oesophagus luminal narrowing and stricture formation. Clinically, adult EoE is characterised by dysphagia due to impaired bolus formation. In children, the disease presents with food refusal, failure to thrive, regurgitation, and vomiting. There is a strong association between EoE and atopy with sensitisation to food allergens, most commonly dairy, eggs, peanuts, fish, wheat, and soy, but elimination diets are effective only in a fraction of these patients [226]. The presence of intraepithelial activated eosinophils is the hallmark of EoE. Eosinophil-derived MBP is crucial to promote mast cell activation, while ECP probably plays a fundamental role in stimulating the secretion of the profibrotic cytokine TGF-*β* from fibroblasts. Since gastroesophageal reflux disease could also lead to damage of the oesophageal epithelium and to oesophageal eosinophilic infiltration, proton pump inhibitor (PPI) therapy is anyway advisable. Specific EoE treatments include elimination diet and oral glucocorticoids [227].

#### 4.3.2. Eosinophilic Gastroenteritis

When eosinophil infiltration into the gastrointestinal tract numerically (>20 per HPF [228]) and functionally exceeds the physiological limits of tissue maintenance (Figure 1), causing clinically relevant symptoms and tissue damage, the term eosinophilic gastroenteritis (EoG) is usually employed. Patients with this rare and heterogeneous disease can be further classified into three different subsets, according to the pattern of eosinophilic infiltration: (a) predominantly mucosal pattern (mucosal and submucosal involvement); (b) predominantly muscular pattern (muscle layers involvement); and (c) predominantly serosal pattern (inflammatory infiltrate reaching the serosal layer). The differential diagnosis with other causes of eosinophilic infiltration includes* Helicobacter pylori* infection, parasitic infections, drug-related adverse events, inflammatory bowel diseases (IBD), connective tissue diseases, and haematological or lymphoid disorders. Although symptoms of EoG are nonspecific and variable, abdominal pain and nausea/vomiting are the most frequent at presentation in children and adults. Children and adolescents can also present with growth retardation, failure to thrive, and delayed puberty or amenorrhea. Atopy and allergic food sensitisation are frequent comorbidities in patients with EoG and imply the need for allergy diagnostics within the disease work-up [228]. The treatment of EoG still lacks a universal standardization but is mainly based on oral courses of corticosteroids. In case of failure on elimination diet (if food sensitisation is found), leukotriene antagonists and disodium cromoglycates represent the second choice treatment [229].

#### 4.3.3. Eosinophilic Colitis and Proctocolitis

Eosinophilic colitis and proctocolitis are inflammatory diseases characterised by eosinophils infiltrating the colonic and/or the rectum wall. Histology can reveal acute inflammation with the characteristic eosinophilic infiltration of the lamina propria (>5 eosinophils per HPF), occasionally in association with lymphoid nodules. These conditions predominantly affect children in their first months of life. IgE-mediated and cell-mediated hypersensitivity to food, in particular to cow milk proteins, constitute the main pathogenic determinants in this population of patients. Allergic proctocolitis manifests with inflammatory changes of the colon and rectum. In exclusively breastfed children, inflammation can be unleashed at time of introduction of cow milk proteins. Symptoms include colic-like symptoms and visible fresh blood mixed with mucus in the stools. The diagnosis is based on the clinical features, whereas the treatment consists in the exclusion of cow milk proteins from patients' and/or their mothers' diet [230].

### 4.4. Eosinophil-Related Respiratory Diseases

#### 4.4.1. Upper Airways

**Figure 3:**
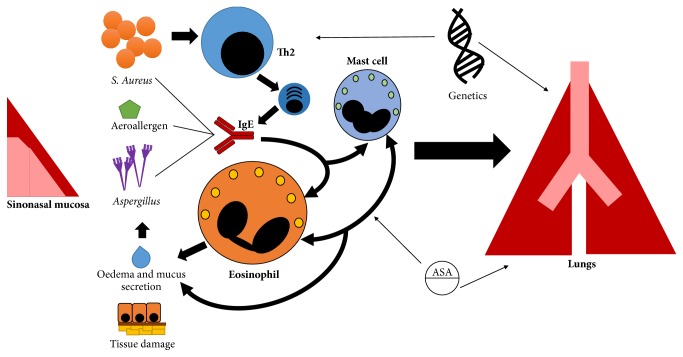
*United airways disease.* Local inflammatory events at the level of the nasal mucosa and paranasal sinuses can correlate with ongoing inflammation in the lungs. Inherited and environmental factors cooperate in the development of such pathological scenario. Eosinophil-dominant responses play a central role in this setting and become active following several stimuli. Conventional allergic responses might prompt mast cell-assisted or direct eosinophil activation, when nasal or paranasal residing cells are challenged with aerial allergens and recognise systemically produced IgE. However, local production of IgE may also occur. Adaptive immune responses can be stimulated by the persistence of inflammatory triggers or by forced superantigenic activation in the setting of* Staphylococcus aureus* colonisation of the mucosal tissues. Disproportionate production of mucosal secretion and loss of tissue integrity due to persistent inflammation may paradoxically favour microbial proliferation and promote further inflammation. A mixture of infectious and allergic features characterises* Aspergillus* colonisation of the paranasal sinuses. Anti-*Aspergillus* IgE account for enhanced eosinophil inflammation. Mast cells are involved in a crucial cross-talk with eosinophils and may dominate the pathogenic cascade in selected conditions such as nonallergic rhinitis with mast cells (NARMA) or eosinophil-mast cell nonallergic rhinitis (NARESMA). Eicosanoids play a major role in this setting as they have vasomotor effects and promote eosinophil recruitment and activation. Nonsteroidal anti-inflammatory drugs may affect this signalling pathway and cause non-IgE-related hypersensitivity reactions at the level of both the upper and lower respiratory tract. In particular, the so-called aspirin exacerbated respiratory disease (AERD) is characterised by a constitutional overproduction of CysLTs, which can be further enhanced by COX-1 inhibitors such acetylsalicylic acid (ASA).

**Figure 4:**
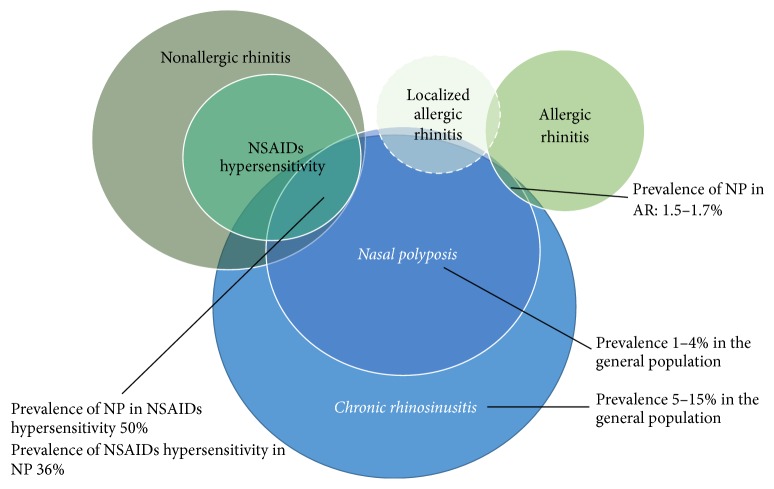
*Prevalence of selected upper airways diseases according to the clinical phenotype.* Several inflammatory diseases of the mucosal layer of the nose and paranasal sinuses may involve eosinophil-related pathogenic events. Taken singularly and together, these entities have a high prevalence in the general population and show multiple overlapping clinical as well as possibly pathophysiological overlaps. In particular chronic rhinosinusitis is very frequent in the general population and associates in a large number of cases with the development of hyperplasia (i.e., nasal polyposis, NP), which in turn is a frequent comorbidity in patients with asthma. Paradoxically to some extent, the prevalence of nasal polyposis in allergic rhinitis (AR) is lower than expected, although atopic patients probably have a worse clinical phenotype. By contrast, nasal polyposis is frequent in patients with nonsteroidal anti-inflammatory drugs (NSAIDs), non-IgE-mediated hypersensitivity, a possible cause of nonallergic rhinitis. However, at least part of the clinical spectrum of nonallergic rhinitis may be characterised by the presence of local IgE responses (LAR). The epistemological borders of this clinical entity are still a matter of debate.

The upper airways constitute a complex set of highly vascularised tissues, which provide a first-line frontier against airborne pathogens and irritants. Accordingly, multiple stressors causing local or systemic alterations in the vascular tone as well in the physiological housekeeping immune response might cause acute or chronic inflammation of the nasal and sinus mucosa and deregulated secretion of mucus [21]. Furthermore, the inflammatory events occurring at the level of the upper respiratory tract can prompt a systemic response, which in turn affects the inflammatory state of the lower respiratory tract, possibly as the expression of an ancestral defensive programme (Figure 3). At a clinical level, when systemic evidence of allergic sensitisation through the detection of specific IgE or skin test reactivity to selected allergens is found, the terms allergic or atopic rhinitis or rhinosinusitis apply. By contrast, the other cases are usually classified as nonallergic or not associated with atopy. However, this nomenclature is possibly misleading since it is limited by the accuracy of the current diagnostic tools for allergy and correlates only partially with the pathophysiology of this group of diseases. In fact, isolated local IgE responses may frequently occur within the sinonasal mucosa and suggest the introduction of novel clinical entities [231] (Figure 4; see also below). In addition, the enhanced mast cell-eosinophil cross-talk constituting the core pathogenic effector of tissue damage in IgE-related disorders is also the pathophysiological hallmark of most so-called nonallergic sinonasal disorders. In fact, local eosinophil infiltrate is consistently, although not invariably, found in either allergic rhinitis, allergic fungal rhinosinusitis, nonallergic rhinitis, or chronic rhinosinusitis with or without nasal polyps. Long established clinical evidence shows that most of these conditions are disproportionately frequent in patients with concomitant asthma, suggesting the existence of a pathophysiological continuum between upper and lower airways inflammation. According to this concept, usually referred to as the “united airways theory,” eosinophil activation in the upper air tract following pharmacological, hormonal, infectious, or environmental stimuli prompts a sustained, possibly IL5-prominent, Th2-driven immune response, which in turn affects the lungs and causes airway hyperresponsiveness and chronic remodelling (Figure 3) [21]. Strikingly, surgical removal of eosinophil-rich pathological tissue within nasal polyps associates with reduced fractional exhaled nitric oxide (FeNO, a surrogate marker of eosinophil inflammation in the lungs) in patients with concomitant asthma [232].

*Allergic Rhinitis.* In allergic rhinitis (AR), the recruitment of eosinophils within the nasal mucosa and lumen is driven by Th2 cytokines and follows the exposure to aeroallergens, in a sensitised subject. Tissue infiltration by eosinophils occurs mainly during the late phase response, which starts 4–6 hours after exposure and lasts for 18–24 hours. An infiltrate containing Th2 cells, eosinophils, and basophils is characteristic of this phase. Th2 cells are important for the production of key cytokines, including IL4, IL5, and IL13. These cytokines are crucial for antibody class switching, regulation of local and systemic IgE synthesis, recruitment of inflammatory cells, and survival of eosinophils. Sensitised subjects have detectable specific IgE able to enhance the degranulation of mast cells and basophils after exposure to the allergen. Auto/exocrine production of IL5 has also a crucial role in this setting. Infiltrating eosinophils release MBP, ECP, and EPO, which in turn injure the nasal epithelial cells and induce nasal hyperresponsiveness to several irritant stimuli.

*Nonallergic Rhinitis.* Nonallergic rhinitis (NAR) is a broad term encompassing heterogeneous forms of rhinitis, characterised by the lack of allergic sensitisation (negative skin testing and/or lack of serum specific IgE) to the aeroallergens implicated in AR (Figure 4). Nasal cytology is essential to differentiate AR from NAR and define NAR subtypes. These are classified according to the dominant cell population and include (1) eosinophilic nonallergic rhinitis (NARES), (2) nonallergic rhinitis with mast cells (NARMA), (3) neutrophilic nonallergic rhinitis (NARNA), and (4) eosinophil-mast cell nonallergic rhinitis (NARESMA). NARESMA has been described as a phenotype of difficult to treat rhinitis [233, 234].

*Local Allergic Rhinitis.* A distinct subset of patients with rhinitis have symptoms suggesting AR, no evidence of systemic atopy assessed by skin prick tests or of serum specific IgE, but show an exacerbation of the rhinitis after nasal challenge. These symptoms are thought to represent the clinical phenotype of an underlying, locally limited, IgE response [235], which, however, can also occur in atopic patients and in healthy subjects [236]. According to the abovementioned clinical definitions, patients with rhinitis without evidence of systemic atopy should fall under the category of NAR. However, some authors claim that the term local allergic rhinitis (LAR) should be applied, when a putative local IgE response (mainly towards house dust-mites and grass/olive pollens [231, 237, 238]) occurs (Figure 4). According to a recent long-term follow-up study, patients with these features can evolve to overt AR in a minority of cases. Nonetheless, the disease seems to have a progressive course and to constitute a potential risk factor for asthma [239].

*Chronic Rhinosinusitis.* Chronic rhinosinusitis (CRS) affects approximately 5–15% of the general population [240]. Only a fraction of CRS patients develop nasal polyps (CRSwNP), suggesting that nasal inflammation could follow different pathogenic pathways [241–243] (Figure 4). NSAIDs hypersensitivity and asthma are part of the united airways disease spectrum and show strong epidemiological and pathogenic associations with CRSwNP (Samter-Widal syndrome). In particular, the term aspirin exacerbated respiratory disease (AERD) refers to a condition characterised by eosinophilic asthma, NSAIDs hypersensitivity, and CRSwNP. Asthma severity has been linked with the risk of relapse in patients with CRSwNP, while surgical treatment of NP ameliorated functional parameters in asthmatic patients [232, 244–246]. Accordingly, FeNO levels are higher in the subset of patients with the more severe phenotype (severe asthma, eosinophilic inflammation, and relapsing polyposis) [232, 245]. These patients are ideal candidate for eosinophil-targeted treatment. Blockade of IL5 showed promising efficacy in patients with CRSwNP and recurrent need for surgery [247]. Anti-IgE therapeutic strategies may also be effective in the setting of persisting LAR and concomitant asthma [248].

#### 4.4.2. Lower Airways Inflammation

*Asthma.* Asthma is a heterogeneous disease that results from the complex interplay between genetic predisposition and environmental factors. Accordingly, several clinical-pathophysiological classifications have been proposed with the ultimate aim of defining asthma endotypes, that is, phenotypes resulting from specific pathogenic mechanisms [249]. Clinically relevant variables include age at disease onset, gender distribution, lung functional parameters, history of atopy, and other concomitant comorbidities such as obesity and smoke and response to corticosteroids and other therapies [250, 251]. In addition, the dichotomy between Th2/eosinophilic and non-Th2/eosinophilic asthma is a cornerstone of asthma classification. In fact, since evidence of eosinophil-driven inflammation can be routinely acquired by means of eosinophil count in blood and sputum as well as by FeNO measurement, sufficient data have been accumulated to support a robust phenotype/endotype correlation between clinically and pathogenically significant Th2/eosinophilic asthma [249]. Clinical evidence suggests that eosinophilia might associate either with atopy-associated forms of early-onset asthma, which show a good response to inhaled corticosteroids (ICS) and bronchodilators, or with severe, nonatopic, recalcitrant forms of late-onset asthma [250]. In particular, a blood cell count greater than or equal to 150 cell/*μ*l under standard of care treatment identifies patients with exacerbation-prone asthma [252]. In addition, eosinophilic airway inflammation, as estimated by sputum cell count and FeNO, increases the risk of eventual virus-induced asthma exacerbation [253]. Notably, high eosinophil counts also independently associate with higher health costs in patients with asthma, irrespectively of asthma severity [254]. However, eosinophil asthma subsets (which also include patients with AERD) account for only half of the asthma population, prompting to the need for further research to define additional endotypes. This task is of outstanding importance since limited response to ICS is a general hallmark of noneosinophilic asthma [255].

Genome-wide association studies (GWAS) suggest that constitutive excessive release of proinflammatory cytokines by the bronchial epithelium and aberrant antigen presentation affect innate as well as adaptive immune responses and have a crucial role for the pathogenesis of asthma [48, 256–258]. In particular, abnormal signalling through IL33 and TSLP (released by the airway epithelium) are thought to promote Th2/eosinophilic responses, whereas altered IL2 and IL18 expression may affect Th1-related responses [256, 257].

The resulting inflammatory patterns are variable and, according to evidence downstream genomics (that is, transcriptomics, proteomics or metabolomics), can be broadly classified in eosinophilic, neutrophilic, paucigranulocytic, and mixed-granulocytic patterns [259]. Eosinophilic inflammation is dominated by ILC-2/Th2-mediated release of IL5 and IL13 and epithelial release of periostin. IL13 and periostin synergise with eosinophil granule moieties and eosinophil-derived TGF-*β* to cause irreversible tissue damage and subsequent remodelling towards fibrosis [260]. Following IgE-restricted or non-IgE-restricted stimuli, mast cells further contribute to airway smooth muscle contraction, eosinophil infiltration, remodelling, and amplification of the inflammatory cascade.

In addition, environmental factors such as drugs, smoke, and microbes may contribute to shape the inflammatory response in patients with asthma [251, 261, 262]. Viral infections account for the majority of asthma exacerbations [253], especially in atopic patients. Conversely, once the T cell-eosinophil axis gets compromised in patients with eosinophilic asthma, a T cell-dependent eosinophil response stereotypically develops when the airways are challenged with viral stimuli [263, 264]. Local host factors may also play a nonnegligible role. Pentraxin 3 (PTX3), a prototypic humoral innate immune mediator with a pleiotropic role in host defence, fertility, and vascular inflammation, seems to play a dual role in this setting, by dampening airway remodelling and stimulating eosinophil inflammation at the same time [265]. Notably, high levels of PTX3 (as well as of anti-PTX3 antibodies) are detectable in patients with superimposed EGPA (see also below), where PTX3 also correlates with disease activity [266, 267]. In this latter setting, PTX3 expression might be enhanced by the concomitance of eosinophilic airway inflammation and small vessel vasculitis [268, 269].

The introduction of biologics for the treatment of asthma prompted an extraordinary improvement in disease control for patients with persistent symptoms under maximal treatment intensity. Omalizumab was first shown to be effective for severe allergic asthma in terms of symptoms control, number of exacerbations, and need for corticosteroids. In addition, it proved able to dampen airway eosinophilic inflammation [82, 270]. Drugs targeting the IL5 pathway have more recently been introduced. In a milestone clinical trial, 100 mg subcutaneous mepolizumab every four weeks was shown to cause a >50% abatement in asthma exacerbation rates in patients with severe eosinophilic asthma [271]. Subsequent studies further confirmed these findings and showed the efficacy of mepolizumab in the amelioration of several asthma-related functional parameters. This evidence has led to the approval of this drug by the European Medicine Agency and the United States Food and Drug Administration for the treatment of severe eosinophilic asthma. Novel anti-IL5 agents such as reslizumab and benralizumab were also able to reduce asthma exacerbations in clinical trials settings [7, 252].

*Nonasthmatic Eosinophil Bronchitis.* Nonasthmatic eosinophil bronchitis (NAEB) is a condition characterised by corticosteroid-responsive cough and eosinophilia >3% at sputum, in the absence of variable airflow obstruction or airway hyperresponsiveness. Eosinophilic asthma and NAEB differ in terms of mast cell localization within the wall of the airways: while in NAEB mast cell infiltration is predominantly epithelial, mast cells from patients with asthma infiltrate the airways smooth muscle and thus account for the typical changes in lung function. The long-term prognosis of NAEB is unknown but a short follow-up study suggests that NAEB does not evolve over time, despite small airway dysfunction increases [272].

*Allergic Bronchopulmonary Aspergillosis.* Diseases related to the ubiquitous airborne fungus* Aspergillus fumigatus* vary depending on the host immune status, immunosuppression being the main risk factor for severe colonisation [131]. Thus, the clinical spectrum ranges from mild respiratory conditions (airways colonisation, IgE-mediated rhinitis, and/or asthma) to severe pulmonary diseases such as allergic bronchopulmonary aspergillosis (ABPA) and chronic pulmonary aspergillosis or invasive aspergillosis. The diagnostic criteria of ABPA are represented in Table 4.* A. fumigatus* triggers Th2-polarised responses, IgE production, lung eosinophilia, and airways hyperresponsiveness [130]. However, in patients with ABPA, a nonresolving and excessive immune response leads also to tissue damage [132]. Recent studies underline that peripheral eosinophil count has limited utility for diagnosing ABPA because it does not correlate with lung function nor with the levels of anti-*Aspergillus* IgE and IgG. Therefore, considering eosinophils count as a minor criterion is a matter of debate. Inversely, the correlation between the peripheral blood eosinophil count and central bronchiectasis and high-attenuation mucus (mucus visibly more dense than the paravertebral skeletal muscle at computed tomography) suggests that eosinophils are one of the primary mediators of inflammation in ABPA [273].

### 4.5. Eosinophilic Granulomatosis with Polyangiitis (EGPA)

Eosinophilic granulomatosis with polyangiitis (EGPA, formerly Churg-Strauss syndrome) is an antineutrophil cytoplasmic antibodies- (ANCA-) associated vasculitis (AAV) that pathogenically lies at the crossroads between asthma, small vessel inflammation, and eosinophil-mediated tissue injury [274, 275]. This complex scenario, combined with the low prevalence of EGPA in the general population [276], has delayed our understanding of the pathophysiological mechanisms underlying the disease and the development of (a) reliable tools for the diagnosis and (b) novel therapeutic options, especially in comparison with other AAVs [80, 277–280]. Of even higher concern, there is still also a lack of universal consensus on the nosologic classification of EGPA. A first set of diagnostic criteria were developed by Lanham and colleagues in 1984 and reflected a unified interpretation of EGPA as a combination of small vessel inflammation, asthma, and eosinophilia [281]. By contrast, the 1990 American College of Rheumatology (ACR) criteria [282] and the pragmatic criteria employed for patients enrolment in the MIRRA study on the efficacy of mepolizumab in EGPA [80] introduced the notion of different disease subsets within the EGPA spectrum. This feature was also comprised in the 2012 Chapel Hill Consensus Conference definition of EGPA, which also stressed the association between positive ANCA and glomerulonephritis [283]. The latest proposal by a joint task force between the French Study Group on Orphan Pulmonary Diseases and the European Respiratory Society (GERMOP/ERS), as a result of a stepwise categorisation approach, suggests more radically to separate a new entity, called hypereosinophilic asthma with (nonvasculitic) systemic manifestations (HASM), from EGPA. According to the GERMOP/ERS diagnostic criteria, only patients with clear features of polyangiitis would thus be diagnosed with EGPA [284] (Table 5). Future studies on the genetic determinants and the pathogenic mechanisms involved in disease development will possibly provide additional support for the refinement of these clinical criteria.

At this regard, currently available evidence clearly indicates a derangement of Th2-related responses as a unifying hallmark of EGPA. However, increasing data support a prominent role of eosinophils as crucial drivers of the disease phenotype. Eosinophils, indeed, are potentially implicated in both the ANCA-unrelated, granulomatous manifestations of EGPA and in the vasculitic, ANCA-related phase of the disease. Specifically, eosinophils can cause direct tissue damage through innate and antibody-dependent mechanisms [285, 286] and cooperate in the maintenance of a Th2-prominent immune response [5, 9, 10, 59]. Furthermore, they might synergise with neutrophils in determining the increased thrombotic risk associated with EGPA [46, 287]. As discussed above, eosinophils also share with neutrophils the ability to generate extracellular DNA traps. However, while NETs are crucial for the induction and maintenance of inflammation in AAV [288] and correlate with eosinophil count in EGPA [289], the potential role of EETs in EGPA is unknown. In addition, little is known about possible genetic factors accounting for enhanced eosinophil activity in EGPA [290], since most candidate-gene studies have focused on the HLA system and on alterations of the adaptive immune response [291]. The results from a GWAS are awaited.

Following the observations made at a pathogenic level, a search for eosinophil-related biomarkers has extensively been performed. Small case-control studies suggested that ECP, IL5, IL25, CCL17, CCL26, and other cytokines are all elevated in patients with active disease and can perform better than conventional markers, including eosinophil count [59, 286, 292–294]. Notably, CCL26 showed promise also in differentiating EGPA from other hypereosinophilic syndromes [290]. However, its reliability as a robust assay to determine disease activity in patients with established diagnosis of EGPA has been questioned by subsequent studies [295].

Eosinophil-targeted therapies constitute the ultimate treatment achievement in EGPA. The 2016 European League Against Rheumatism (EULAR) guidelines for the management of AAV recommend a combination of glucocorticoids plus cyclophosphamide or rituximab to induce remission in new-onset or relapsing, organ-threatening, or life-threatening AAV and a combination of glucocorticoids plus methotrexate or mycophenolate mofetil for remission-induction in non-organ-threatening AAV [75]. In all these cases, a lower grade of evidence for EGPA is acknowledged, although growing evidence is accumulating [296], in particular on the use of rituximab in the induction and maintenance phase [278, 297, 298]. The results of the first randomised placebo-controlled trial on the efficacy of mepolizumab in refractory or relapsing non-organ- or life-threatening EGPA have been recently published. Mepolizumab, administered at a dosage of 300 mg every four weeks (three times the dosage employed for eosinophilic asthma) in addition to standard of care (glucocorticoids and immunosuppressants excluding cyclophosphamide or biologics), proved clearly superior over placebo for remission-induction and for the prevention of further relapses with no increased rates of adverse events. Nonetheless, remission did not occur in 47% of patients in the mepolizumab arm (versus 81% in the placebo arm), pointing again to the need for further studies to better dissect different disease subtypes among the EGPA spectrum, which could be amenable for different treatments [80]. Additional studies are currently ongoing to assess the efficacy of other eosinophil-targeted therapies such as benralizumab (NCT03010436) or reslizumab (NCT02947945) in EGPA.

## 5. Conclusion

Eosinophils play a crucial role in the immune homeostasis both as effector immune cells committed to host defence and as modulators of the shape of innate and adaptive immune responses. Furthermore, they are involved in the control of the functional homeostasis of several nonimmunocompetent tissues and possibly in tissue repair. An intricate, eosinophil-centred, signalling network comprising Th2 lymphocytes, B cells, and mast cells as well as circulating platelets and cells residing at sites of inflammation is activated under inflammatory stimuli to ensure host protection from parasitic, fungal, bacterial, and viral infections. However, the same mechanism accounts for the development of tissue damage during infections, clonal diseases of the eosinophils, and/or of eosinophil-related cell subsets as well as in hypersensitivity reactions and autoimmune diseases. Thanks to recent development in our understanding of these pathogenic events, several eosinophil-targeted therapies are currently under development in preclinical or clinical scenarios and offer promising perspectives for the future treatment of eosinophil-mediated diseases.

## Figures and Tables

**Table 1:** Functional characterisation of eosinophil granules.

*Primary granules *		

Galectin 10 (CLC protein)		Charcot-Leyden crystals formation in tissues and fluids lysophospholipase activity Potential immunoregulatory function towards T cells

*Specific/crystalloid granules*		

Crystal core	MBP	Disrupts lipid layers and increases membrane permeability → cytotoxic to host cells and pathogens Component of EETs Basophils, neutrophils & mast cells activation and degranulation Neuroprotective effect Epithelial activation and expression of tissue remodelling factors Increases smooth muscle reactivity Inhibits M2 muscarinic receptors

Matrix	EDN	(potent) RNAse → antiviral role (ssRNA viruses) Neurotoxicity (Purkinje cells) Dendritic cells chemotaxis, maturation and activation → proliferation of T and B cells
	ECP	(Weak) RNAse Cytotoxic to host cells and pathogens (parasites, viruses, bacteria) Neurotoxicity (Purkinje cells) Membrane disruption Component of EETs
	EPO	Generation of ROS toxic to extracellular pathogens (helminth parasites, bacteria) Pro- and anti-inflammatory effects Epithelial activation and expression of tissue remodelling factors Lipid peroxidation

*Lipid bodies *		

	Arachidonic acid derivatives (LT, PG, TX)	Promotion of acute and late hypersensitivity responses Prominent role in airways inflammation

ECP: eosinophil cationic protein; EDN: eosinophil derived neurotoxin; EETs: eosinophil extracellular traps; EPO: eosinophil peroxidase; LT: leukotrienes; MBP: major basic protein; PG: prostaglandins; ROS; reactive oxygen species; TX: thromboxanes.

**Table 2:** Clonal disorders with primary eosinophilia.

Disease	Most common associated mutations/rearrangements	Diagnostic features
Myeloid/lymphoid neoplasms with eosinophilia and abnormalities of PDGFRA, PDFRB, FGFR1, or PMC1-JAK2.	Involvement of: (i) 4q12 (platelet-derived growth factor receptor alfa) (ii) 5q31–q33 (platelet-derived growth factor receptor beta) (iii) 8p11-12 (fibroblast growth factor receptor 1) (iv) 9p24 (Janus kinase 2)	Eosinophilia *and* positive FISH or molecular screening in PDGFRA, PDGFRB, FGFR1, PMC1-JAK2

Chronic myeloid leukaemia (CML) BCR-ABL+	t(9;22)(q34.1;q11.2) (B cell receptor–Abelson)	BCR-ABL positive at molecular screening, t(9;22) in cytogenetic analysis

Systemic mastocytosis (SM)	(KIT D816V mutation)	Mast cells increased in marrow aspirate and biopsy, KIT mutation, tryptase increased

Chronic eosinophilic leukaemia not otherwise specified (CEL, NOS)	Possible involvement of TET2, ASXL1, IDH2, JAK2, SETBP1, SF3B1, EXH2, CBL	Eosinophilia *and* non-specific clonal or molecular abnormalities and/or increased marrow blasts

Acute myeloid leukaemia with inv(16)	Inv(16)(p13.1,1q22) or t(16;16)(p13.1;q22)	>20% myeloblasts on marrow aspirate/biopsy *and* positive cytogenetic/FISH analysis

Lymphocyte-variant hypereosinophilia (L-HES)	T cell receptor clonality	Abnormal T-cell immunophenotype *and/or* demonstration of clonal TCR rearrangement by molecular biology

**Table 3:** Diagnostic criteria for DRESS.

RegiSCAR criteria (three or more required)	J-SCAR (seven or more required):
(i) Hospitalization (ii) Reaction suspected to be drug related (iii) Fever > 38°C (iv) Acute skin rash (v) Hematologic abnormalities (eosinophilia, atypical lymphocytosis, low platelets) (vi) Lymphadenopathy (vii) Internal organ involvement	(i) Maculopapular rash developing > 3 weeks after drug exposure (ii) Prolonged clinical symptoms after discontinuation of the causative drug (iii) Fever > 38°C (iv) Liver abnormalities (ALT > 100 U/l) or other organ involvement (v) Lymphadenopathy (vi) WBC abnormalities (≥1) (vii) Leukocytosis (viii) Atypical lymphocytes (ix) Eosinophilia (x) HHV-6 reactivation

**Table 4:** Diagnostic criteria for ABPA [23].

Hierarchy of criteria	Criteria for ABPA diagnosis
*Major criteria* Both necessary, but not sufficient	Allergic sensitisation to A fumigatus: positive skin prick test or detectable or raised sIgE to *A. fumigatus*
	Elevated total IgE levels > 1000 kIU/l (=2400 *μ*g/l)

*Minor criteria* Two or three are sufficient	*Aspergillus* IgG serology positive or raised, detection of anti-*Aspergillus* precipitins
	Radiology, according to the clinical stage: transient migratory pulmonary opacities to fixed central bronchiectasis
	Elevated circulating eosinophils > 0.5 or 1 × 10^9^/l

***Background***	Asthma, cystic fibrosis

**Table 5:** Nosologic classification of EGPA.

Chapel Hill Consensus Conference 2012 definition of EGPA			

Eosinophil-rich and necrotizing granulomatous inflammation often involving the respiratory tract, and necrotizing vasculitis predominantly affecting small to medium vessels, and associated with asthma and eosinophilia. ANCA is more frequent when glomerulonephritis is present			

Classification and diagnostic criteria			

Lanham's (1984) diagnostic criteria	ACR (1990) classification criteria	MIRRA (2017) classification criteria	GERMOP/ERS (2017) diagnostic criteria

Mandatory criteria (i) Asthma (ii) Blood eosinophilia > 1500/mm^3^ or >10% of total WBC (iii) Evidence of vasculitis involving two or more organs	Criteria (at least 4/6 required) (i) Asthma (ii) Blood eosinophilia > 10% of total WBC (iii) Neuropathy (iv) Pulmonary infiltrates nonfixed (v) Paranasal sinus abnormalities (vi) Extravascular eosinophils	Mandatory criteria: (i) *Asthma* (ii) *Eosinophilia* (>1.0 × 10^9^/L and/or >10% of total blood leucocytes) Minor criteria (at least two required): (i) *Positive biopsy* showing histopathological evidence of eosinophilic vasculitis, or perivascular eosinophilic infiltration, or eosinophil-rich granulomatous inflammation. (ii) *Neuropathy:* either mononeuritis or polyneuropathy demonstrated by a motor deficit or nerve conduction abnormality (iii) (nonfixed) *pulmonary infiltrates* (iv) *Sino-nasal abnormality* (v) *Cardiomyopathy *at echocardiography or cardiac magnetic resonance imaging (vi) *Glomerulonephritis* (vii) *Hematuria*, red cell casts, proteinuria (viii) *Alveolar haemorrhage*, confirmed by bronchoalveolar lavage (ix) *Palpable purpura* (x) *Positive ANCA* (MPO or PR-3)	Entry criteria for EGPA and HASM (i) Asthma (ii) Blood eosinophils ≥ 1.5 × 10^9^/l or ≥10% of total WBC *Eosinophilic granulomatosis with polyangiitis (EGPA)* Criteria (at least 1/4 required): (i) Definite vasculitis features (a) Biopsy-proven necrotising vasculitis of any organ (b) Biopsy-proven necrotising glomerulonephritis or crescentic glomerulonephritis (c) Alveolar haemorrhage (d) Palpable purpura (e) Myocardial infarction due to proven coronaritis (ii) Definite surrogates of vasculitis (a) Haematuria associated with red casts or >10% dysmorphic erythrocytes or haematuria and 2+ proteinuria on urine analysis (b) Leukocytoclastic capillaritis and/or eosinophilic infiltration of the arterial wall at biopsy (iii) Mononeuritis or mononeuritis multiplex (iv) ANCA and any systemic manifestation *Hypereosinophilic asthma with systemic manifestations (HASM)* Mandatory criteria (i) Any systemic manifestation other than definite polyangiitis or surrogate of vasculitis or mononeuritis (ii) Absence of ANCA
